# 4D treatment planning for scanned ion beams

**DOI:** 10.1186/1748-717X-2-24

**Published:** 2007-07-03

**Authors:** Christoph Bert, Eike Rietzel

**Affiliations:** 1 Gesellschaft für Schwerionenforschung (GSI), Abteilung Biophysik, Planckstraße 1, 64291 Darmstadt, Germany

## Abstract

At Gesellschaft für Schwerionenforschung (GSI) more than 330 patients have been treated with scanned carbon ion beams in a pilot project. To date, only stationary tumors have been treated. In the presence of motion, scanned ion beam therapy is not yet possible because of interplay effects between scanned beam and target motion which can cause severe mis-dosage. We have started a project to treat tumors that are subject to respiratory motion. A prototype beam application system for target tracking with the scanned pencil beam has been developed and commissioned.

To facilitate treatment planning for tumors that are subject to organ motion, we have extended our standard treatment planning system TRiP to full 4D functionality. The 4D version of TRiP allows to calculate dose distributions in the presence of motion. Furthermore, for motion mitigation techniques tracking, gating, rescanning, and internal margins optimization of treatment parameters has been implemented. 4D calculations are based on 4D computed tomography data, deformable registration maps, organ motion traces, and beam scanning parameters.

We describe the methods of our 4D treatment planning approach and demonstrate functionality of the system for phantom as well as patient data.

## Background

### Intrafractional target motion

Intrafractional target motion has a relevant impact on the precision of treatment delivery in conformal radiotherapy. Even if treatment planning margins are sufficient to encompass the full extent of motion, intrafractional motion degrades dose gradients to surrounding healthy tissue [1,2]. For intensity modulated therapy like IMRT or scanned particle therapy, the relative motion between target and multi-leaf collimator or scanned beam can have a severe impact on the delivered dose. These interplay effects between target and beam motion usually cause hot and cold spots in the delivered dose distributions [1,3-10].

To mitigate the impact of intrafractional motion, several techniques were proposed but have not yet been used clinically with scanned particle beams: beam gating [11,12], rescanning [13,14], tracking [15-17], and internal margins (IM) to generate an internal target volume (ITV) [18]. Besides tracking, all other techniques require IMs due to unmitigated or residual motion. For particle beams, the use of ITVs requires explicit consideration of possible range changes because ranges are most often influenced by organ motion [19,20]. Adjustments of the beam range, e.g. by compensator smearing, have to be applied to ensure coverage of the distal field edge for all motion states of the target [19,21].

Precise motion mitigation techniques require quantification of the motion, for example by time resolved computed tomography (4DCT) [22-26] or 4D magnetic resonance tomography (MRT) [27]. Both techniques sample periodical motion in several motion phases. The motion phases correspond to quasi-static 3D volumes, e.g. standard CT volumes. Usually sampling is based on an external motion surrogate [28-30]).

Several investigators have used time-resolved volumetric imaging to extend treatment planning capabilities for tumor sites influenced by respiratory motion [21,31-34]. The main idea was reported by Keall et al. [32] and Rietzel et al. [33]: Dose calculations are performed per motion phase of 4DCT data. Deformation maps obtained by non-rigidly registering motion phases are then used to transform the resulting sub-dose distributions to a reference motion phase for effective dose calculation by time weighted summation.

### Carbon ion therapy at GSI

At Gesellschaft für Schwerionenforschung (GSI) charged particle therapy is performed with an intensity-modulated, raster-scanned carbon ion beam in collaboration with the University Hospital Heidelberg, the German Cancer Research Center, and the Forschungszentrum Rossendorf. To date, more than 330 patients with tumors in the head, neck, spinal cord, and pelvis region have been stereotactically treated within the pilot project [35,36]. GSI plans to treat tumors affected by respiratory motion by tracking. Consequently we have started a project to develop beam application and treatment planning capabilities for this technique [15,37,38]. For a short introduction, the following paragraphs summarize standard treatment delivery and treatment planning for scanned carbon ion beams as well as the current status of the tracking project.

Treatment delivery is performed with the active raster-scanner system [39]. In beam's eye view, the planning target volume (PTV) is divided into *j *slices of iso-energies *E*_*j *_(typical slice distance: 3 mm water-equivalent). Each iso-energy slice contains a regular grid (typical grid spacing: 2–3 mm) of *i *beam positions (*x*_*i*_, *y*_*i*_). Individual pencil beams with a focus of 3–9 mm (full width at half maximum) are applied per grid position. To achieve the desired dose distribution, the number of carbon ions *N*_*ij *_is optimized for each position. During treatments, a synchrotron accelerates carbon ion pencil beams to the required beam energy *E*_*j*_. Particle extraction from the synchrotron is performed in beam pulses with a length of 2.2 s followed by 3.3 s of acceleration for the next energy. For each iso-energy slice (IES) a new pulse has to be requested from the synchrotron because beam energy can – up to now – not be changed within a pulse. For each IES the pencil beam is scanned by a magnetic deflection system across all beam positions with *N*_*ij *_> 0. The scanning process is controlled by fluence monitors. They measure the number of particles deposited per beam position and request transition to the next position as soon as the required number of particles *N*_*ij *_has been reached. The beam is not turned off during transition to the next grid position. Scanning speed (up to 11m/s) is thus dependent on *N*_*ij *_and the synchrotron extraction profile.

Treatment planning is performed with the GSI treatment planning system TReatment planning for Particles (TRiP) based on a native computed tomogram (CT) and a set of contours [40,41]. Dose calculation is performed with a pencil beam algorithm. For calculation of dose *D *at each CT voxel center (*x*, *y*, *z*) the contributions from all beam positions are summed:

D(x,y,z)[Gy]=1.6⋅10−8∑energies jgrid pos. id(Ej,z)[MeVg cm-2]Nij2πσ2[mm2]exp⁡(−(x−xi)2+(y−yi)22σ2)
 MathType@MTEF@5@5@+=feaafiart1ev1aaatCvAUfKttLearuWrP9MDH5MBPbIqV92AaeXatLxBI9gBaebbnrfifHhDYfgasaacH8akY=wiFfYdH8Gipec8Eeeu0xXdbba9frFj0=OqFfea0dXdd9vqai=hGuQ8kuc9pgc9s8qqaq=dirpe0xb9q8qiLsFr0=vr0=vr0dc8meaabaqaciaacaGaaeqabaqabeGadaaakeaacqWGebarcqGGOaakcqWG4baEcqGGSaalcqWG5bqEcqGGSaalcqWG6bGEcqGGPaqkdaWadaqaaiabbEeahjabbMha5bGaay5waiaaw2faaiabg2da9iabigdaXiabc6caUiabiAda2iabgwSixlabigdaXiabicdaWmaaCaaaleqabaGaeyOeI0IaeGioaGdaaOWaaabuaeaacWaSaoizaqMamalGcIcaOiadWc4GfbqrdGaSaUbaaSqaialGcWaSaoOAaOgabKaSacGccWaSakilaWIamalGdQha6jadWcOGPaqkdGaSaoWaaeacWc4aialGlaaabGaSakadWcyGnbqtcWaSagyzauMamalGbAfawbqaialGcWaSag4zaCMamalGbccaGiadWcyGJbWycWaSagyBa02aialGCaaaleqcWcyaialGcWaSagyla0IamalGbkdaYaaaaaaakiacWcOLBbGaialGw2faamacWc4caaqaialGcWaSaoOta40aialGBaaaleacWcOamalGdMgaPjadWc4GQbGAaeqcWciaaOqaialGcWaSaIOmaidcciGamalG=b8aWjadWc4FdpWCdGaSaYbaaSqajalGbGaSakadWciIYaGmaaGcdGaSaoWaaeacWcOamalGb2gaTjadWcyGTbqBdGaSaYbaaSqajalGbGaSakadWcyGYaGmaaaakiacWcOLBbGaialGw2faaaaac4aSakyzauMamalGcIha4jadWcOGWbaCdGaSagWaaeacWcOamalGgkHiTmacWc4caaqaialGdGaSagWaaeacWcOamalGdIha4jadWcOHsislcWaSaoiEaG3aialGBaaaleacWcOamalGdMgaPbqajalGaaGccGaSaAjkaiacWcOLPaaadGaSaYbaaSqajalGbGaSakadWciIYaGmaaGccWaSaA4kaSYaialGbmaabGaSakadWc4G5bqEcWaSaAOeI0IamalGdMha5nacWc4gaaWcbGaSakadWc4GPbqAaeqcWciaaOGaialGwIcacGaSaAzkaaWaialGCaaaleqcWcyaialGcWaSaIOmaidaaaGcbGaSakadWciIYaGmcWaSa+3Wdm3aialGCaaaleqcWcyaialGcWaSaIOmaidaaaaaaOGaialGwIcacGaSaAzkaaaalqaabeqaaiabbwgaLjabb6gaUjabbwgaLjabbkhaYjabbEgaNjabbMgaPjabbwgaLjabbohaZjabbccaGiabdQgaQbqaaiabbEgaNjabbkhaYjabbMgaPjabbsgaKjabbccaGiabbchaWjabb+gaVjabbohaZjabb6caUiabbccaGiabdMgaPbaabeqdcqGHris5aaaa@1E2A@

*d*(*E*_*j*_, *z*) quantifies the energy loss distribution for a certain beam energy *E*_*j *_with respect to traversed amount of tissue *z *in water-equivalent units. During optimization, the energy levels *E*_*j *_and the raster grid (*x*_*i*_, *y*_*i*_) are set to cover the extent of the PTV in beams-eye-view. The minimal beam-width is determined by the grid-spacing (uniform in *x *and *y*) according to *σ *> 1.27 (*x*_*i *+ 1 _- *x*_*i*_). To calculate the longitudinal extent (*z*_min _- *z*_max_) of the target, CT numbers are converted into particle ranges based on a Hounsfield look-up table [42]. Optimization of *N*_*ij *_is performed by least square minimization such that *D*(*x*, *y*, *z*) meets the prescribed dose.

**Figure 1:**
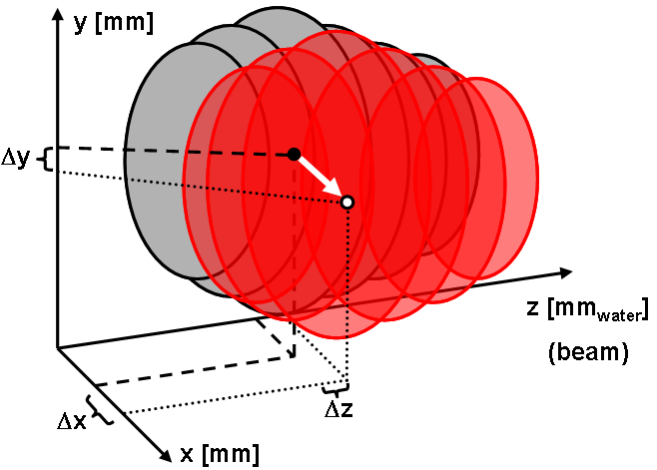
**Schematic drawing of 3D tumor motion in the coordinate system of the scanned ion beam**. The tumor is depicted in its reference position (grey) and in a second position corresponding to a different motion phase (red). The scanning coordinate system consists of iso-energy layers (*z *in water-equivalent units) as well as grid positions within these layers (*x*, *y*). Motion of a grid position is indicated by a motion vector (white). Δ(*x*, *y*, *z*) are the parameters required for motion compensation.

**Figure 2:**
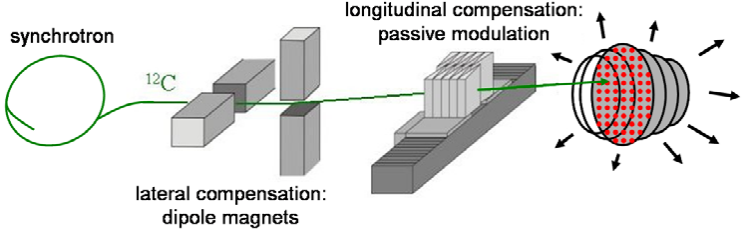
**Schematic outline of the prototype compensation system developed at GSI**. For each iso-energy slice, carbon ions are accelerated in a synchrotron to the required energy *E*. Laterally, the pencil beam is scanned by magnetic deflection. Lateral compensation is performed by adapting the nominal magnet settings. Longitudinal compensation has to be performed with a pair of plastic wedges mounted on linear motors because the beam energy can currently not be changed during an extraction cycle. Particle ranges are adapted by varying the thickness of the traversed plastic in the beam. (image according to [37])

For tracking, beam parameters (*x*, *y*, *z*) have to be adjusted at the time of irradiation to compensate motion of the target (see fig. 1). A prototype system has been built which allows lateral and longitudinal adaptation of the beam (see fig. 2) [37]. In beam's eye view, adaptation of the lateral beam position (Δ*x*, Δ*y*) is performed by changing the target positions of the raster-scanner system during delivery. Changes in particle range Δ*z *have to be compensated with a passive energy modulation system because synchrotron settings can not yet be adapted within an extraction pulse. In our current prototype system a pair of lucite wedges mounted on linear motors is used [43].

### 4D treatment planning for scanned ion beams

The combination of scanned particle beams and target motion represents a double-dynamic system that requires a dedicated solution for 4D treatment planning. We extended our treatment planning system TRiP [40] to full 4D functionality to allow dose calculation and parameter optimization in the presence of motion. In principle the 4D functionality can handle all types of motion. In the following we will, however, focus on respiratory motion as this is our initial intended application.

In order to compare different motion mitigation techniques like tracking, gating, rescanning, and internal margins, the 4D version of TRiP allows to

i. generate particle specific ITVs for treatment plan optimization of gating and rescanning,

ii. optimize parameters for motion compensation (tracking),

iii. calculate physical dose distributions in the presence of motion as well as for tracking, gating, rescannning, and the use of internal margins.

In contrast to previous simulation studies of our group [15,17], we have implemented 4D treatment planning based on multiple volumetric data sets, e.g. CT data sets. In addition, ITV generation and optimization of tracking parameters including methods to correct for target rotation and deformation were realized. Calculation of physical dose distributions can be performed for patient data with patient specific, non-rigid motion.

The purpose of this contribution is the technical description of the 4D treatment planning extensions. The functionality will be presented for phantom simulations as well as for an example patient data set. Experimental validation and application to clinical data of lung cancer patients will be reported elsewhere.

## Dose calculations in the presence of target motion

For scanned particle beams, 4D dose calculations require temporal correlation of beam motion and organ motion considering possible changes in particle range. Dose calculations are based on a reference motion phase independent of the motion mitigation technique (see fig. 1). The following sections describe the calculation of dose distributions in the presence of motion as well as the parameters that are required for these calculations.

### Organ motion parameters

For treatment planning we assume organ motion to be non-rigid as well as represented by time-resolved volumetric data that allows precise calculation of particle ranges, e.g. CT data. For respiratory motion measured by 4DCT, motion is assumed to be periodical. Temporally, organ motion has to be measured in correlation to beam motion (section Beam motion parameters). Spatially, organ motion is described with respect to the particle beam and with respect to a reference motion phase (fig. 1).

3D information of the motion like amplitude, trajectory, and volumetric changes are included in the 4DCT phases. Quantitatively, the motion is parametrized by B-splines which describe the non-rigid motion components between 4DCT phases. Optimization and application of these transformation maps are performed with *vtkCISG *[44]. Details on calculation and validation of the transformation data have been reported previously by Rietzel et al. [33,45].

Temporal changes from 3D data set to 3D data set were implemented via motion traces. For example 4DCT period and initial respiratory phase can be given by a motion trajectory. The 4D version of TRiP allows handling of measured motion trajectories, modeling of sinusoidal motion, or modeling motion according to Lujan et al. [46].

### Beam motion parameters

With beam motion we refer to the time dependent movement of the raster-scanned pencil beam as it traverses the target volume grid-position by grid-position and slice-by-slice (see fig. 2 and section Carbon ion therapy at GSI). Pencil beam motion is quantitatively determined by the particle intensity profile extracted from the synchrotron, the number of particles per grid-position *N*_*ij*_, the order of beam positions (*x*_*i*_, *y*_*i*_) within each iso-energy slice *j*, and the order in which iso-energy slices are irradiated.

Particle extraction is not exactly deterministic or reproducible (see fig. 2 in [15]). Slight changes in the acceleration and especially the extraction process lead to changes that do not affect irradiations of stationary targets but cause changes in the temporal progress of the scanning process. For precise dose calculations in the presence of organ motion, beam intensity and irradiation time of each individual beam position (typical duration < 10 ms per position) have to be considered in temporal correlation to organ motion.

The 4D version of TRiP can handle measured intensity distributions and can model the extraction characteristics at GSI as well as the characteristics of the Heidelberg Ion-Therapy center (HIT, under construction) [47]. In contrast to GSI's synchrotron with so called slow extraction, knock-out-extraction [48] will be used at HIT. This extraction method allows intermitted extraction within one pulse and thus optimal gated irradiations. Modeling of the extraction pattern for gated irradiations has to be performed for each motion trajectory and gating window combination individually because the pulse structure (~1.5s acceleration for each iso-energy slice followed by a maximal pulse length of 10 s) is fixed. Treatment planning can thus also be used to estimate realistic treatment times for gated irradiations.

### Generation of quasi-static sub-treatment plans

For all motion mitigation techniques, treatment delivery is based on a reference motion phase. For respiratory motion, this reference motion phase typically corresponds to the end-exhale phase of the 4DCT data. Using the standard functionality of TRiP [40], a reference treatment plan (*x*, *y*, *E*, *N*) is optimized on the reference motion phase. This reference treatment plan is then applied to the moving target by raster-scanning. The reference treatment plan is modified by compensation parameters Δ(*x*, *y*, *z*, *N*) at time of delivery for tracking, interrupted for delivery by gating, applied multiple times for rescanning, and unchanged for mitigation by internal margins.

**Figure 3:**
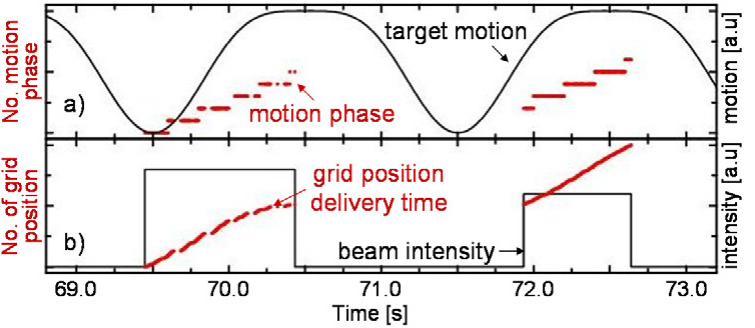
**Temporal correlation of scanning progress and organ motion**. Dose calculation requires temporal correlation of scanning progress and organ motion. a) Based on a motion trajectory the actual motion phase is determined. In this example 10 motion phases were used. b) Scanning progress is determined by the extracted beam intensity and the number of particles per grid position *N*_*ij*_. Scanning progress, i.e. the delivery time of each grid position, is not linear because *N*_*ij *_can differ within iso-energy slices (left). Signal heights are plotted in arbitrary units.

Independent of the motion mitigation technique, 4D dose calculations have to temporally correlate the delivery of each single pencil beam position with the motion of the target as described by motion trajectory and 4DCT (see fig. 3b). In analogy to 4DCT, the reference treatment plan is split into sub-treatment plans by attributing each individual grid position to a motion phase. This is performed by processing motion characteristics simultaneously to beam extraction profiles. Then each sub-treatment plan includes all beam positions (*x*, *y*, *E*, *N*) of the reference treatment plan which are irradiated during the corresponding motion phase (see fig. 3a). Within our treatment planning code, splitting of treatment plans can be based on motion amplitude or phase of the motion trajectory. Ideally, it should be consistent with the method used during 4DCT data acquisition. Then sub-treatment plans and corresponding 4DCT phases can be used to calculate sub-dose distributions. Each sub-dose distribution contains the cumulative dose delivered by the reference treatment plan during that specific motion phase – over all occurrences of that motion phase during the treatment.

In contrast to other motion mitigation techniques, tracking changes beam parameters. Corresponding sub-treatment plans require consideration of compensation parameters. Adjustment of lateral beam positions (Δ*x*, Δ*y*) and numbers of particles Δ*N *can readily be applied (e.g. *x*_new _= *x *+ Δ*x*). The longitudinal change Δ*z *corresponds to a shift of the depth dose distribution (*d*(*E*, *z *+ Δ*z*)) and has to be considered in the summation of dose contributions (eq.1).

### Effective dose distributions

Sub-dose distributions represent the cumulative dose delivered within a specific motion phase. For evaluation of a treatment scenario (e.g. mitigation technique, target motion parameters, extraction rate), the effective dose distribution of the complete treatment plan has to be calculated. Because the quasi-static motion phases are not anatomically registered the sub-dose distributions can not simply be summed but need to be transformed to the reference motion phase. Transformation maps which quantitatively describe the non-rigid motion (see section Organ motion parameters) are used to transform each sub-dose distribution to the reference motion phase. The transformed sub-dose distributions can then be summed time weighted to calculate the effective dose distribution.

### Dose calculation examples

**Figure 4:**
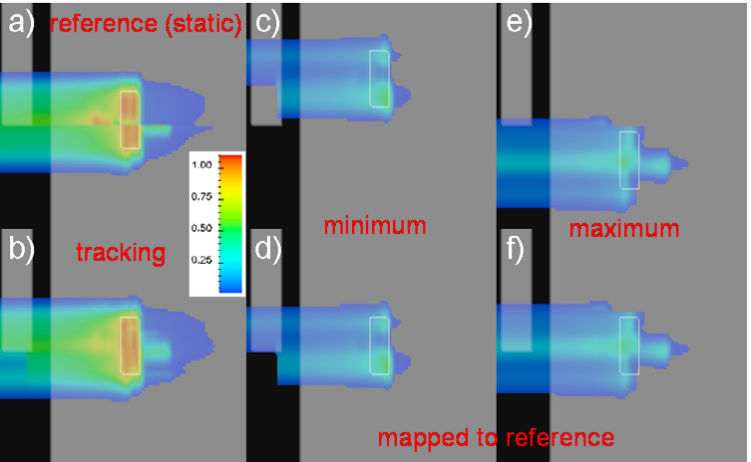
**Simulation of a phantom irradiation**. Simulation of a phantom irradiation to illustrate the steps of dose calculation in the presence of motion using tracking. 4DCT data were modelled static, with exception of the target (white contour) which moves up and down. Shown are overlays of relative dose distributions on the CT motion phases. a) Reference motion phase with reference dose distribution for the stationary target. b) The resulting dose distribution for tracking which is comparable to a). Small differences distal of the sharp CT gradient occur due to the finite pencil beam width (see text for details). The effective dose distribution is the weighted sum of transformed sub-dose distributions at all motion phases. Sub-dose distributions at extreme target positions are shown in c) and e) and transformed to the reference motion phase in d) and f).

To illustrate the steps of 4D dose calculation, we present the irradiation of a simple phantom with tracking in fig. 4. The phantom is homogeneous except for the slab on the left top with higher density. A 4DCT data set was constructed with the indicated target volume (white contour) moving periodically. A treatment plan was optimized to deposit a homogeneous dose distribution in the target on the reference 4DCT phase (fig. 4a). In addition, a sinusoidal motion trajectory was constructed. The temporal correlation of target motion and scanned beam leads to sub-treatment plans and quasi-static sub-dose distributions for each motion phase. Fig. 4c, e show the sub-dose distributions for peak motion phases comparable to end-inhalation and end-exhalation. Despite the motion the dose is deposited in the target volume because tracking compensates target motion including adaptation of particle ranges (shift of *d*(*E*, *z*) visible in fig. 4c). Fig. 4d, f show the same sub-dose distributions transformed to the reference motion phase. The effective, summed dose distribution on the reference motion phase is shown in fig. 4b. It is comparable to the reference dose distribution calculated for a stationary target in fig. 4a. The only differences occur distal of the sharp CT gradient caused by the slab on the left top. The width of the pencil beams (~7 mm full width at half maximum) results in overshooting distal of the sharp gradient for some parts of the diameter of the pencil beams (this is an artificial situation which is unlikely to occur in patient treatment). For motion tracking, this effect is largely reduced, i.e. smeared out, because the static CT gradient causes the described effect at different positions in the moving target volume.

## Optimization of treatment plans

Changes in the optimization of treatment plans for moving targets in comparison to the algorithms used for standard irradiations (see section Carbon ion therapy at GSI and [40]) depend on the motion mitigation technique. The following sections describe our implementations of rescanning, gating, internal margins, and tracking.

### Optimization for rescanning, gating, and internal margins

In contrast to tracking, gating and rescanning do not require change of treatment parameters during delivery. However, particle specific ITVs have to be generated for both techniques. Particle specific ITVs account for possible range changes due to organ motion [21]. ITVs ensure target dose coverage in each 4DCT phase for rescanning or for the subset of 4DCT phases included in the gating window.

Timing of the beam delivery sequence is modified for gating, where the beam is turned on only within the gating window, e.g. close to the end-exhale breathing phase. The gating window has to be defined for the optimization. Rescanning also changes timing of the beam delivery sequence because the same irradiation pattern is applied several times. The number of particles per grid position is therefore divided by the number of rescans. Typically the beam fluence is constant but the scanning speed increases. Besides ITV generation, the definition of the number of rescans is the main task for optimization of rescanning.

The procedure for ITV generation in our active beam delivery environment is as follows:

i. Required input: native 4DCT and ITV contour

ii. Calculation of the maximal lateral ITV extension in beam's eye view (BEV) and setup of the raster grid (*x*_*i*_, *y*_*i*_)

iii. Definition of iso-energy slices (IES with accelerator energies *E*_*j*_): The IES are arranged from the distal to the proximal water-equivalent extension of the ITV. Because tumor motion influences particle ranges the extreme water equivalent ranges of all 4DCT phases have to be considered per grid position to ensure target dose coverage to the distal edge independent of the motion phase during irradiation.

iv. Calculation of the water-equivalent depth for each CT voxel as required for dose calculation (*d*(*E*, *z*) in eq.1): Because the depth is influenced by organ motion the maximum depth from all 4DCT phases has to be used per voxel.

v. Optimization of *N*_*ij *_at each grid position (*x*_*i*_, *y*_*i*_) based on the maximum water-equivalent depth data to achieve the required dose distribution.

**Figure 5:**
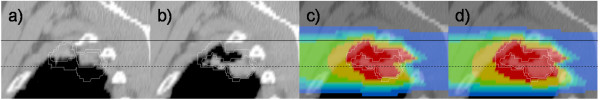
**ITV optimization**. The effect of optimization to an ITV incorporating 4DCT information. Reference 4DCT phase at end-exhalation (a) and end-inhalation (b) with GTV and ITV contours in white. c) If treatment plan optimization to the ITV is based on the reference 4DCT phase only, dose coverage of GTV_inhale _can not be guaranteed. d) GTV_inhale _coverage is achieved by including range information of all 4DCT phases into the optimization of the ITV based treatment plan. The dose distributions in c) and d) are calculated for a stationary target and end-inhalation.

The difference between ITV generation with and without consideration of 4DCT range information is demonstrated in fig. 5 for a lung tumor patient. Fig. 5a displays the 4DCT reference phase at end-exhalation with contours of GTV_exhale _and ITV, the end-inhalation motion phase is shown in fig. 5b. If optimization of a treatment plan to the ITV is based on the reference 4DCT phase only, dose coverage of the distal GTV_inhale _(stationary target at end-inhalation) edge is not sufficient (fig. 5c). Incorporating range information from all 4DCT phases results in adequate coverage (fig. 5d).

The additional treatment parameters for gating and rescanning (gating window and number of rescans) can be determined or even optimized by performing corresponding dose calculations. Simulated organ motion trajectories and particle extraction rate are necessary for this step. By variation of the parameters for organ motion and beam application coverage of the CTV can be analyzed for different gating and rescanning parameters.

### Optimization of tracking-parameters

#### Treatment delivery by tracking

For tracking, target motion is mitigated by adaptation of the reference treatment plan parameters during treatment delivery. Calculation of the compensation parameters Δ(*x*, *y*, *z*) is based on a 4DCT data set and corresponding transformation maps (cf. section Organ motion parameters). Compensation parameters have to be calculated during treatment planning because calculations are too time-consuming to be performed online during treatment delivery. Because motion trajectory and temporal scanning progress (determined by the synchrotron extraction) are not known at the time of treatment planning, compensation parameters have to be calculated for all possible interplay combinations. During treatment delivery, the motion phase is continuously measured, ideally with the same system as used for 4DCT acquisition. Based on the currently irradiated grid position the corresponding pre-calculated compensation parameter set Δ(*x*, *y*, *z*) is used for beam adaptation. Fluctuations in synchrotron extraction have no impact on compensation parameter sets because the intensity controlled raster-scanning process determines which grid-position is irradiated. The motion detection system continuously determines the actual motion phase or interrupts the irradiation if the current motion state is not included in the pre-calculated parameter sets.

**Figure 6:**
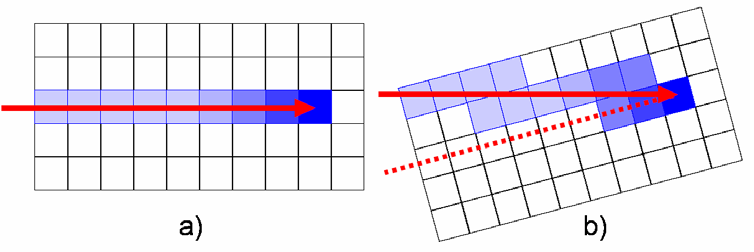
**Impact of non-translational motion components on dose deposition**. a) Reference dose distribution: Irradiation of a beam position (arrow) results in dose deposition along the beam path (color gradient). b) For motion including rotations or deformations simple motion compensation by adaptation of the Bragg peak position is not sufficient because dose deposition in the entrance channel changes.

Adaptation of the 3D pencil beam position Δ(*x*, *y*, *z*) only is not sufficient if organ motion includes non-translational degrees of freedom. In general, irradiation of a specific grid position results in dose deposition at nearby as well as more proximal grid positions (see fig. 6a). These dose contributions are considered during optimization of the reference treatment plan. For simple translational motion, the optimized dose distribution can be achieved by tracking because beam adaptation compensates translations and the 3D grid of pencil beam positions is unchanged. For non-translational degrees of freedom, e.g. rotations or deformations, the 3D grid position arrangement changes. As a consequence dose contributions from nearby or more distal grid positions change in comparison to the initial reference treatment plan (see fig. 6b). For particles prone to fragmentation such as C-12, an additional, similar effect occurs. The so called fragmentation tails will also be deposited at different positions in comparison to the reference, but with smaller doses compared to the ones deposited in the entrance channel.

Mitigation of dose changes Δ*D *due to dose contributions that differ from those in the reference treatment plan can be achieved by adaptation of the number of particles Δ*N *at each 3D grid position. Because the target is scanned only once in a pre-determined manner, Δ*D *depends on the irradiation order of iso-energy slices and the scan path within each slice. Dose contribution mitigation is only possible for dose changes resulting from grid positions irradiated previously. The irradiations should therefore start with the highest beam energy at the most distal slice because dose contributions to different grid positions are significantly higher in the entrance channel than in the fragment tail.

#### Calculation of compensation parameters

The number of required beam position compensation parameters Δ(*x*, *y*, *z*) is determined by the number of grid positions and the number of motion phases because in principle each grid position can be irradiated during each motion phase. The detailed calculation of the compensation parameter combinations is as follows:

i. Determination of the CT coordinate of each grid position in the reference treatment plan by conversion from the water-equivalent system to the CT system based on the reference 4DCT phase (fig. 1, grey volume)

ii. Motion vector determination (fig. 1): the transformation maps (section Organ motion parameters) provide the geometrical transformation into all other 4DCT phases.

iii. Lateral compensation (Δ*x*, Δ*y*): corresponds to the motion vector components

iv. Longitudinal compensation component Δ*z*: corresponds to the change in particle range between the original grid position in the reference 4DCT phase and the transformed grid position (fig. 1, white circle in red volume) in the corresponding 4DCT phase.

**Figure 7:**
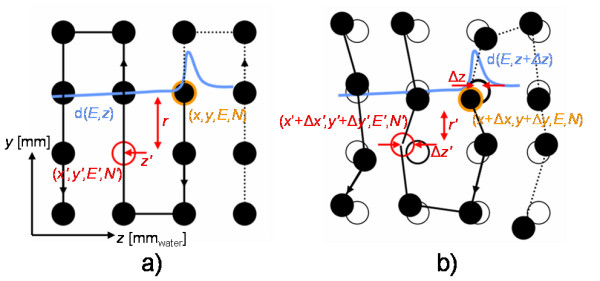
**Dose contribution compensation**. a) For the reference motion phase dose contributions from each grid position (*x*, *y*, *E*, *N*) on grid positions irradiated later (*x'*, *y'*, *E'*, *N'*) are calculated based on lateral distance *r *and depth *z'*. b) Changes in dose contribution Δ*D *are computed based on the 4DCT phase of interest including the adaptation of Bragg peak position Δ(*x*, *y*, *z*) and Δ(*x'*, *y'*, *z'*). This results in a shift of the depth dose distribution *d*(*E*, *z *+ Δ*z*), shift of *z' *(by Δ*z'*), as well as a change in lateral distance (*r'*).

To compensate for variations in dose contributions, the dose change Δ*D *is determined as part of optimization for each beam position and each motion phase. Since each grid position causes specific dose contributions to other grid-positions and since these contributions depend on the actual motion phase during irradiation, resulting dose changes depend on the interplay pattern. Both, under- and over-dosage in comparison to the reference treatment plan are possible. The steps to calculate parameters for dose contribution compensation are as follows:

i. For each grid position of the reference treatment plan dose contributions to all grid positions irradiated afterwards are determined on the reference 4DCT phase (see fig. 7a).

ii. The calculation in (i) is repeated for all possible motion phases. This dose calculation has to consider the changed 4DCT phase as well as the compensation based on the Bragg peak position, Δ(*x*, *y*, *z*) (fig. 7b).

iii. The change in dose Δ*D *due to different contributions is the difference between the dose contributions of (i) and (ii).

During treatment, changes in deposited dose Δ*D *are used to determine the required change in particle deposition Δ*N*. Each grid position causes dose changes Δ*D *at several other grid-positions irradiated afterwards which are taken from the pre-calculated data depending on the motion phase valid at the time of delivery. Consequently each grid position suffers from dose changes from grid-positions irradiated previously. For determination of Δ*N*, the cumulative dose changes from all previously irradiated grid positions is used (∑Δ*D*). The adjustment Δ*N *is given by Δ*N *= -*ND*^-1^∑Δ*D *where *N *and *D *are parameters of the reference treatment plan. If *N *+ Δ*N *is negative, i.e. if too much dose has been applied, no particles are delivered at the grid position but the over-dosage can not be corrected for. This shows the lack of optimization for the described approach, but this is unavoidable if arbitrary motion trajectories of beam and target can occur. The main goal is avoidance of under-dosage.

#### Example patient data

**Figure 8:**
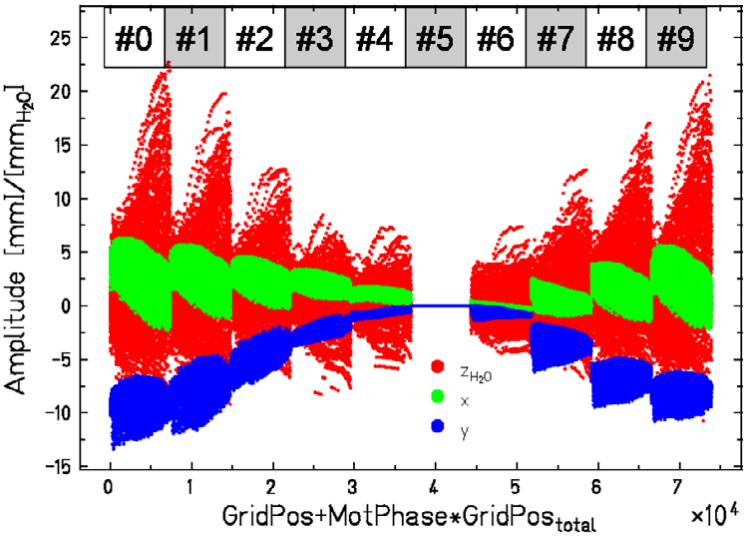
**Motion compensation parameters**. For all combinations of reference treatment plan grid positions (GridPos, (*x*, *y*, *E*, *N*)) and motion phases (MotPhase #0 – #9) a compensation vector Δ(*x*, *y*, *z*_water_) is computed. In this example the reference plan consisted of 7389 grid positions (GridPos_total_). For 10 motion phases, this results in 73890 compensation vectors to describe beam adaptation from the reference motion phase (#5) to all other 9 motion phases. Parameters are shown for an anterior-posterior field, so the *y *component in the scanning coordinate system (beam's eye view) corresponds to cranio-caudal motion. For this patient, peak-to-peak tumor motion amplitude was approximately 12 mm.

The 4DCT data of the lung tumor patient shown in fig. 5 were used to compare different motion mitigation techniques. Apart from ITV generation (fig. 5 and section Optimization for rescanning, gating) the calculation of compensation parameters for tracking was necessary. The 4DCT consists of 10 motion phases. The phase corresponding to end-exhalation was used as reference 4DCT phase. The reference treatment plan for tracking to the CTV consisted of 7389 grid positions. Thus 73890 beam position compensation vectors were calculated, each with two lateral components (Δ*x*, Δ*y*) in millimeter and a longitudinal component Δ*z *in millimeter water-equivalence. The resulting data are shown in fig. 8. The reference treatment plan was optimized for an anterior-posterior field, so the *y*-component of the scanner coordinates (up-down in beam's eye view) corresponds to cranio-caudal motion, which was on the order of 12 mm peak-to-peak.

**Figure 9:**
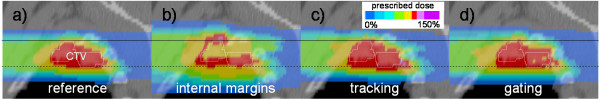
**4D treatment planning for a lung tumor**. Dose distributions (color-wash) on the reference 4DCT phase (grey-levels, CTV contour in white) for different motion mitigation techniques in comparison to (a) the dose distribution for a stationary target. (b) Internal margins can not properly mitigate target motion. Interplay between beam scanning and organ motion leads to a deteriorated dose distribution. Tracking (c) and gating (d) allow appropriate mitigation of target motion. Resulting dose distributions cover the CTV comparable to (a). Motion parameters: respiratory period 2 s, initial respiratory phase 90°, Lujan model (n = 2), synchrotron extraction modeled for HIT.

The results from dose calculations for the reference treatment plan on the end-exhalation phase as well as for the mitigation techniques internal margins, tracking, and gating are shown in fig. 9. In comparison to the reference dose distribution (stationary, fig. 9a), tracking (fig. 9c) and gating (fig. 9d) allow comparable CTV coverage in the presence of motion. Fig. 9b shows that internal margins can not be used to mitigate motion influence for a scanned beam because interplay between target motion and beam motion prevails.

## Discussion

We have implemented 4D treatment planning for charged particle radiotherapy with scanned pencil beams. Dose calculations in the presence of motion as well as optimizations for gating, rescanning, and tracking are based on time-resolved anatomical data, motion trajectories, and extraction characteristics of the accelerator.

Theoretically, tracking of the target with the scanned pencil beam should result in the best possible sparing of surrounding, healthy tissues. Whereas for gating and rescanning, required ITV margins lead to an increased PTV that encompasses surrounding tissues. For gating, the size of the gating window and the resulting residual target motion will determine ITV expansions. Then a trade off between treatment delivery time and target conformity has to be made. Comparing the motion mitigation techniques, it should be noted that increasing conformity will elevate technical complexity. As long as tracking has not been developed for clinical routine use, we clearly favor gating in comparison to rescanning due to the increase in conformity. Currently, treatment planning studies based on 4DCT patient data are performed to explore and quantitatively evaluate the differences between motion mitigation techniques.

In general, treatment planning – independent whether in 3D or 4D – relies on the validity of the input data. In current practice, these data are most often assumed to be ground truth throughout the treatment course. Generation of the required input data is out of the scope of this paper. We will therefore only briefly discuss possible implications on 4D treatment planning.

4DCT samples moving anatomy in several discrete 3D motion phases. Usually motion is detected during data acquisition by an external monitoring system to sort CT data according to respiratory phases. As pointed out by several authors, 4DCT is not free of residual motion artifacts for example due to irregular respiration [22,23,25,26,49]. Such artifacts will have an impact on 4D planning, manifested by wrong position of the target and possible changes in particle ranges. Possible improvements in 4DCT data acquisition are currently under investigation [50-52].

Target motion trajectories could be measured directly by fluoroscopy [53,54] or indirectly by external motion detection systems [28-30]. Techniques and limitations of motion detection systems are out of the scope of this contribution; we assume a reliable detection of motion phases. For retrospective dose calculations, motion trajectories have to be recorded during irradiations only. For optimization of motion mitigation strategies, the treatment delivery system has to react to actual motion phases online. Fluoroscopic tracking has been successfully used in Japan [53]. Treatments are gated with millimeter precision based on trajectories of fiducial markers close to the target. Currently, fluoroscopic tracking of the target or nearby structures without fiducial markers is under investigation. If external motion detection systems have to be used, ideally, the same motion detection system would be used during treatment delivery as was used for 4DCT data acquisition.

Besides target motion beam motion during scanned beam application has to be considered to model interplay effects. Recording of the irradiation time of each beam position has already been implemented at GSI. Treatment times for individual pencil beam positions are typically below 10 ms which usually results in less than 0.1 mm of motion for typical respiratory parameters.

## Conclusion

We extended GSI's treatment planning system TRiP to full 4D functionality. The new modules facilitate 4D dose calculation and optimization for tracking, gating, rescanning, and internal margins. Calculations and optimizations are based on 4DCT information, organ motion, and trajectory of the scanned ion pencil beam.

## Competing interests

The *Moving Targets *project at GSI is partially funded by Siemens Medical Solutions, Particle Therapy. ER is now employed by Siemens Medical Solutions, Particle Therapy.

## Authors' contributions

Both authors contributed equally to the design of the methods and algorithms. Implementation was performed by CB.
